# Inhibition of miR-25 attenuates doxorubicin-induced apoptosis, reactive oxygen species production and DNA damage by targeting PTEN

**DOI:** 10.7150/ijms.41980

**Published:** 2020-06-05

**Authors:** Zhiqiang Li, Hongqiang Li, Baoxin Liu, Jiachen Luo, Xiaoming Qin, Mengmeng Gong, Beibei Shi, Yidong Wei

**Affiliations:** Department of Cardiology, Shanghai Tenth People's Hospital, Tongji University School of Medicine, 301 Middle Yanchang Road, Shanghai, 200072, China

**Keywords:** H9c2 cells, doxorubicin-induced cardiotoxicity, miR-25, PTEN

## Abstract

**Background**: Doxorubicin (DOX) is one of the widely used anti-cancer drugs, whereas it can induce irreversible cardiac injury in a dose-dependent manner which limits its utility in clinic. Our study aimed to investigate the relationship between miR-25 and DOX-induced cardiac injury and its underlying mechanism.

**Methods**: Mice and H9c2 cells were exposed to DOX. The overexpressed or knockdown of miR-25 in H9c2 cells was achieved by miR-25 mimic or inhibitor and the efficiency of transfection was identified by qRT-PCR or Western blotting. Cell viability, apoptotic cell rate, and levels of apoptosis-related proteins were determined by CCK-8, flow cytometry, and Western blotting, respectively. Furthermore, Western blotting and immunofluorescence staining (IF) were performed to assess the expression levels of reactive oxygen species and degree of DNA damage.

**Results**: As a result, DOX significantly upregulated miR-25 expression in mice and H9c2 cells and reduced cell viability and increased cell apoptosis *in vitro* and *in vivo*. miR-25 overexpression expedited cell injury induced by DOX in H9c2 cells demonstrated by the increased cell apoptosis and reactive oxygen species (ROS) production, whereas miR-25 inhibition attenuated the cell injury. Furthermore, miR-25 negatively controlled the expression of phosphatase and tensin homolog deleted on chromosome 10 (PTEN). Intervention the expression of PTEN using si-PTEN reversed the beneficial effects of miR-25 inhibition on DOX-injured H9c2 cells.

**Conclusion**: In conclusion, this study demonstrated that miR-25 is involved in DOX-induced cell damage through the regulation of PTEN expression.

## Introduction

Doxorubicin (DOX) is an effective anthracycline chemotherapy agent in treating pediatric and adult neoplasms. However, the progressive and dose-related cardiotoxicity induced by DOX impedes its clinical use, which might render the happen of acute cardiotoxicity or chronic heart failure after years of latency 1-4. As the relative survival rate of cancer patients has been improved, more attention is paid on DOX-induced cardiomyopathy. Previous studies demonstrated that DOX-induced cardiotoxicity was mainly related to the generation of excess reactive oxygen species (ROS), iron accumulation, DNA damage, autophagy and thus cell death 5-8. However, clinical trials using antioxidants failed in delaying the process of heart failure after treated with DOX 9. Accordingly, further exploration of the molecular mechanism of DOX-induced cardiotoxicity, such as microRNAs (miRNAs), is warranted in finding out some new treatment targets.

MiRNAs are an important category of non-coding RNAs (ncRNAs) of 19-24 nucleotides that modulates several cellular processes, such as cell differentiation, proliferation and survival, mainly via binding to the 3′ untranslated region (UTR) of mRNAs, resulting in translational repression, degradation or deadenylation 10, 11. Emerging evidence has revealed that miRNAs are involved in the process of DOX-induced cardiotoxicity, such as miR-34a-5p, miR-29b, and miR-140-5p 12-14. MicroRNA-25 (miR-25), a member of the miR-106b-25 cluster whose host gene (MCM7 oncogene) located on chromosome 7q22.1, played an important role in numerous biological process 15. Recently, it was observed that miR-25 was altered in the process of cardiovascular diseases 16-18. Overexpression of miR-25 induced heart hypertrophy and injection of an antisense oligonucleotide (antagomiR) against miR-25 markedly halted established heart failure in a mouse model, improving cardiac function and survival compared with a control antagomiR oligonucleotide, and these effects were further enhanced when using miR-25 Tough Decoy inhibitors 16, 17. Moreover, the deletion of miR-106b~25 cluster could increase sarcoplasmic reticulum (SR) Ca^2+^-leak in atrial and thus resulting in atrial fibrillation (AF) by increasing the expression of Ryanodine Receptor Type-2(RyR2) 18. However, the function of miR-25 in DOX-induced cardiac injury has not been reported yet.

The aim of this study was to investigate the effects and the potential mechanism of miR-25 in DOX-induced cardiac injury. We found that the level of miR-25 was increased after DOX treatment, whereas the expression of phosphatase and tensin homolog deleted on chromosome 10 (PTEN) decreased. Overexpression of miR-25 using miR-25 mimic could aggravate DOX-induced apoptosis, ROS and thus DNA damage in H9c2 cells. Reversely, miR-25 inhibitor could alleviate these damages made by DOX. The potential mechanism underlying it might be attribute to its regulation on PTEN expression.

## Materials and Methods

### Animals and treatment

Adult male C57BL/6 mice (~8-10 weeks, 20-25g) obtained from the Vital River Laboratory Animal Technology Co. Ltd. (Beijing, China) were randomly assigned to the DOX-treat group and the control (CTRL) group with weight and age matched (n=7 for each group). DOX (Solarbio, D8740, Beijing, China) or saline was injected via intraperitoneal (i.p.) at a dose of 5 mg/kg repeated every week for four times (cumulative dose:20mg/kg) 19. One week after treatment, echocardiography assessment was performed and then the hearts were harvested and fixed in 4% paraformaldehyde (PFA) overnight for further study. The hearts were cut into 5μm slices and stained with haematoxylin and eosin (HE). Animal experiments were approved by the Animal Care and Use Committees of Shanghai Tenth People's Hospital and in accordance with the Guide for the Care and Use of Laboratory Animals (National Institutes of Health Publication No. 85-23, revised 1996).

### Echocardiography

After mice were anesthetized by isoflurane, echocardiography was conducted blindly using a Vevo2100 imaging system (VisualSonics Inc, Toronto, ON, Canada) as previously described^20^. M-mode images of the left ventricle were recorded at the papillary muscle level when the heart rate (HR) of the mice was maintained at 450-500 bpm. The fractional shortening (FS), left ventricular ejection fraction (EF), left ventricular end-diastolic dimension (LVEDD) and left ventricular end-systolic dimension (LVESD) were measured. Imaging and calculations were carried out by an experienced technician who was unaware of the treatment applied to each animal.

### Cell culture and transfection

The H9c2 cells, HEK293T cells and diffuse large B-cell lymphoma (DLBCL) cell lines (NUDUL-1 and TMD8) were all purchased from American Type Culture Collection (ATCC, Manassas, VA, USA). H9c2 cells and HEK293T cells were cultured in Dulbecco's modified Eagle's medium (DMEM, HyClone, Logan, USA) containing with 10% fetal serum (FBS, Gibco, Gaithersburg, USA) and 1% penicillin-streptomycin solution. NUDUL-1 and TMD8 cells were cultured in Roswell Park Memorial Institute (RPMI) 1640 medium (Gibco). miR-25 mimic/NC mimic, miR-25 inhibitor/NC inhibitor and PTEN siRNA/NC siRNA were synthesized by Ribobio Technology Co, Ltd (Guangzhou, China). After seeded into plates at indicated density, cells were transfected with these agents for 24-48 hours by Lipofectamine 2000 (Invitrogen, CA, USA) referring the protocol of manufacture. The transfection efficacy was determined by RT-PCR or Western blot. After transfection, cells were exposed to 5μM DOX for 24 hours and harvested for further experiments. The primer of siRNA for PTEN is as follow: GGCTAAGTGAAGACGACAA.

### Cell viability assay

The cell viability was determined by Cell Counting Kit-8 (CCK-8, Dojindo, Japan) in accordance with the manufacturer's protocol. Cells were seeded into the 96-well plates and exposed to indicated concentration of DOX for variable time. Then, the optical density (OD value) was measured by a Microplate Reader (SpectraMax 190, Molecular Device, USA) at 450nm. Cell viability was calculated as the percentage of absorbance, comparing treated cells with untreated cells and repeated with three times.

### Apoptosis analysis

Cell apoptosis were determined by Annexin V-FITC Apoptosis Dectection Kit (BD Biosciences, San Diego, CA). Briefly, cells were collected and washed twice with chilled phosphate-buffered saline (PBS). Then cells were centrifuged with 900 rpm for 5 minutes and resuspend with 500μl binding buffer. Annexin V-FITC (5μl) and propidium iodide (PI) (5μl) were added into the solution for 15 minutes at room temperature in the dark. Finally, cells were analyzed by a flow cytometer (BD Biosciences, San Jose, CA, USA). A Terminal transferase UTP nick end labelling (TUNEL, Roche, Mannheim, Germany) assay was also performed to detect apoptosis. Cells were incubated with nucleotide polymers for 1 hour and detected by Olympus IX83 microscope (OLYMPUS, Japan). The percentage of apoptotic cells were calculated by number of TUNEL-positive cells/total cells×100%.

### Measurement of reactive oxygen species (ROS) and antioxidant activity

The intracellular formation of ROS was assayed by oxidative conversion of cell-permeable 2'7'-dichlorofluorescein diacetate (DCFH-DA, Beyotime Biotechnology, Shanghai, China) to fluorescent DCF. Following indicated treatment, cells were wash with PBS by twice and introduced by 10μM DCFH-DA in serum-free DMEM in dark for 30 min at 37℃. After washing with serum-free DMEM by twice, the DCF fluorescence intensity was measured with a fluorescent microscope. The superoxide dismutase (SOD), catalase (CAT) and glutathione peroxidase (GSH-Px) assay kit, purchased from Bioengineer Company (Nanjing, China) were used to measure the SOD, CAT and GSH-Px activities according to the manufacturer's instructions, respectively.

### Immunofluorescence staining (IF)

H9c2 cells were seeded on coverslips and fixed with 4% PFA for 20 minutes at room temperature after treatments. Then, the cells were permeabilized using 0.1% Triton X-100 solution for 20 minutes and blocked with 3% BSA for 30 minutes. After incubation with primary antibody against γ-H2AX (Abcam, Cambridge, MA, USA, 1:100 diluted) and α-SMA (Abcam, 1:100 diluted) overnight at 4°C in a humidified chamber, the cells were covered with ProLong® Gold Antifade Reagent with DAPI (Invitrogen) and visualized with a fluorescence microscope.

### Quantitative real-time polymerase chain reaction (qRT-PCR)

Total RNA was extracted from left ventricular myocardial tissues or H9c2 cells by TRIzol reagent (Life Technologies, Shanghai, China) according to manufacturer's instruction. cDNA was synthesized using the PrimeScript RT reagent kit (Takara Biotechnology Ltd, Dalian, Liaoning, China) from 2 μg of RNA and detected with SYBR Premix EX TaqII (Takara, Japan) using Light Cycler 96 (BIOTECON Diagnostics, Roche, Switzerland). MiRNA quantification was determined by the Bulge-loopTM miRNA qRT-PCR Primer set (one RT primer and a pair of qPCR primers for each set) specific for miR-25, which was designed and synthesized by RioboBio Co. Ltd with U6 as internal control to normalize gene expression using the 2^-ΔΔCT^ method. Cycle threshold (C_T_) were defined as the point when the fluorescence signal got a predefined threshold value (the back ground level). ΔC_T_ is equal to the difference in the threshold value for target genes and internal control, and ΔΔC_T_ is calculated as the difference in the threshold value of ΔC_T_ between the treatment group and control group: ΔΔC_T_= (C_T Dox-miR-25_ - C_T Dox-U6_ ) - (C_T Control-miR-25_ - C_T Control-U6_) 21.

### Luciferase reporter assay

The 3′-untranslated region (3′-UTR) sequence of PTEN containing predicted binding sites of miR-25 was obtained by PCR and inserted into the pMIR-REPORT luciferase reporter vectors (Promega, Fitchburg, MA) to get the constructs and referred as PTEN wild-type (PTEN-WT). Meanwhile, the mutation predicted binding sites was also inserted into the pMIR-REPORT luciferase reporter vectors, which were referred as PTEN mutant-type (PTEN-MUT). During Luciferase analysis, HEK293T cells were transfected with PTEN-WT+miR-25 inhibitor, PTEN-MUT+miR-25 inhibitor, PTEN-WT+NC inhibitor and PTEN-MUT+NC inhibitor respectively using Lipofectamine 2000 reagent (Invitrogen). The luciferase activity was determined using Dual Luciferase Assay (Promega, Madison, WI) 48 hours after transfection.

### Western blot analysis

After proper treatment, cells were lysed with lysis buffer (Cell Signaling Technology, CST, MA, USA) containing 1mM PMSF and centrifuged at 15,000 rpm for 20 min. The supernatant was recollected and quantified. 30μg proteins were used from each sample and electrophoresed on 10% SDS-PAGE gels. After transferred onto polyvinylidene fluoride (PVDF) membranes (Millipore Corporation, MA, USA), the membranes were blocked with 5% non-fat milk and then incubated with primary antibodies overnight at 4˚C. The following primary antibodies were used: anti-Bcl-2 (Abcam), anti-Bax (CST), anti-PTEN(CST), anti-phosphatidylinositol 3 kinase (PI3K, CST), anti-p-PI3K (CST), anti-protein kinase B (AKT, CST), anti-p-Akt (CST), anti-γ-H2AX (Abcam), anti-β-actin (CST) and anti-glyceraldehyde-3-phosphate-dehydrogenase (GAPDH, CST). Subsequently, members were washed by TBST (150 mM NaCl, 50 mM Tris pH 7.5, 0.1% Tween-20) and incubated with the appropriate secondary antibodies for one hour at room temperature for one hour. The blots were scanned with ECL Western Blotting Detection Reagent (Tanon, Shanghai, China) by Amersham Imager 600 ECL system (GE Healthcare, USA).

### Statistical analysis

Data are presented as the mean ± standard error of mean (SEM) and analyzed using a statistical software (SPSS 20.0; Chicago, IL, USA). A comparison between the control and treatment group was performed using the unpaired Student's t-test. Differences among the groups were determined by one-way analysis of variance (ANOVA) followed by Tukey's post-hoc test. P<0.05 was considered to indicate a statistically significant difference.

## Results

### DOX increases the expression of miR-25 in H9c2 cells

**Figure 1:**
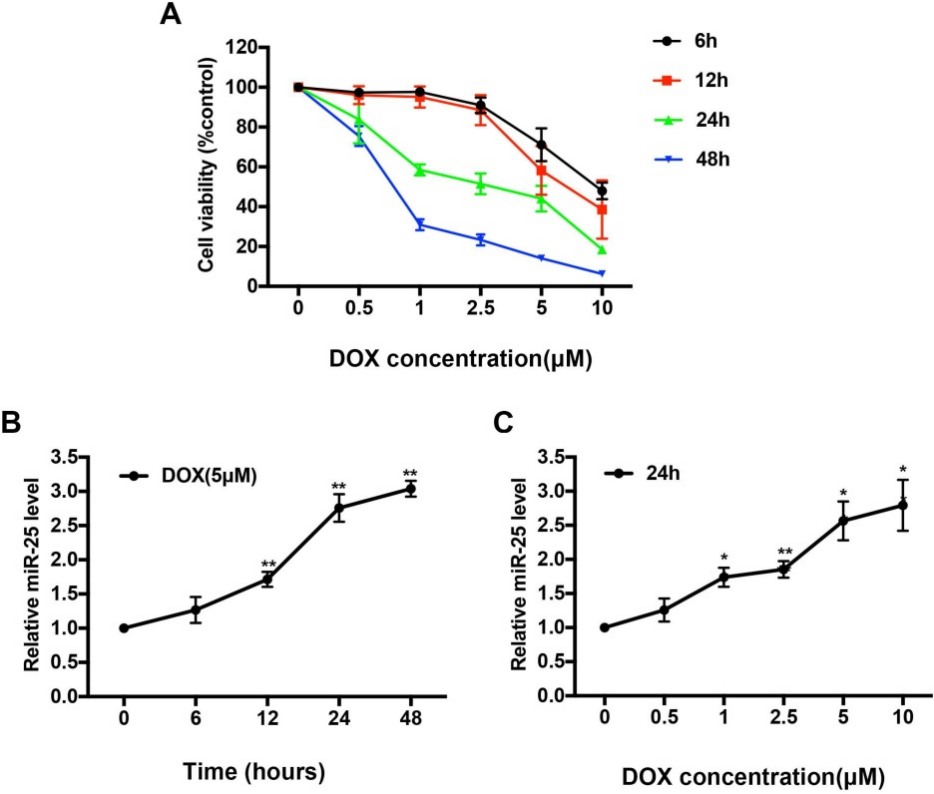
** DOX upregulates the level of miR-25 in H9c2 cells.** (A) CCK8 assay shows the reduced proliferation of H9c2 cells treated with increased concentration of DOX for 6, 12, 24, and 48h. Effect of exposure to (B)5μM DOX for different time points and (C) different concentration of DOX for 24h on the expression of miR-25 determined by qRT-PCR in H9c2 cells. (*P < 0.05; **P < 0.01, compared with cells in 0h, n=4)

H9c2 cells, which derived from embryonic rat heart tissue, own many characteristics of cardiomyocytes. To access the cytotoxicity of DOX, we exposed H9c2 cells to various concentrations of DOX (0, 0.5, 1, 2.5, 5 or 10μM) for 0, 6, 12, 24, or 48 hours respectively. As shown in Figure 1A, the cell viability of H9c2 was significantly decreased by around 50% in 5μM after DOX treatment for 24 hours, so we chose this condition for further* in vitro* study. Expression level of miR-25 was significantly increased in a time- and dose-dependent manner confirmed by qRT-PCR upon treatment with DOX (Figure 1B and 1C), suggesting that miR-25 is involved in DOX-induced cardiomyocyte injury.

### DOX induces cardiac injury and upregulates the level of miR-25 in mice

**Figure 2:**
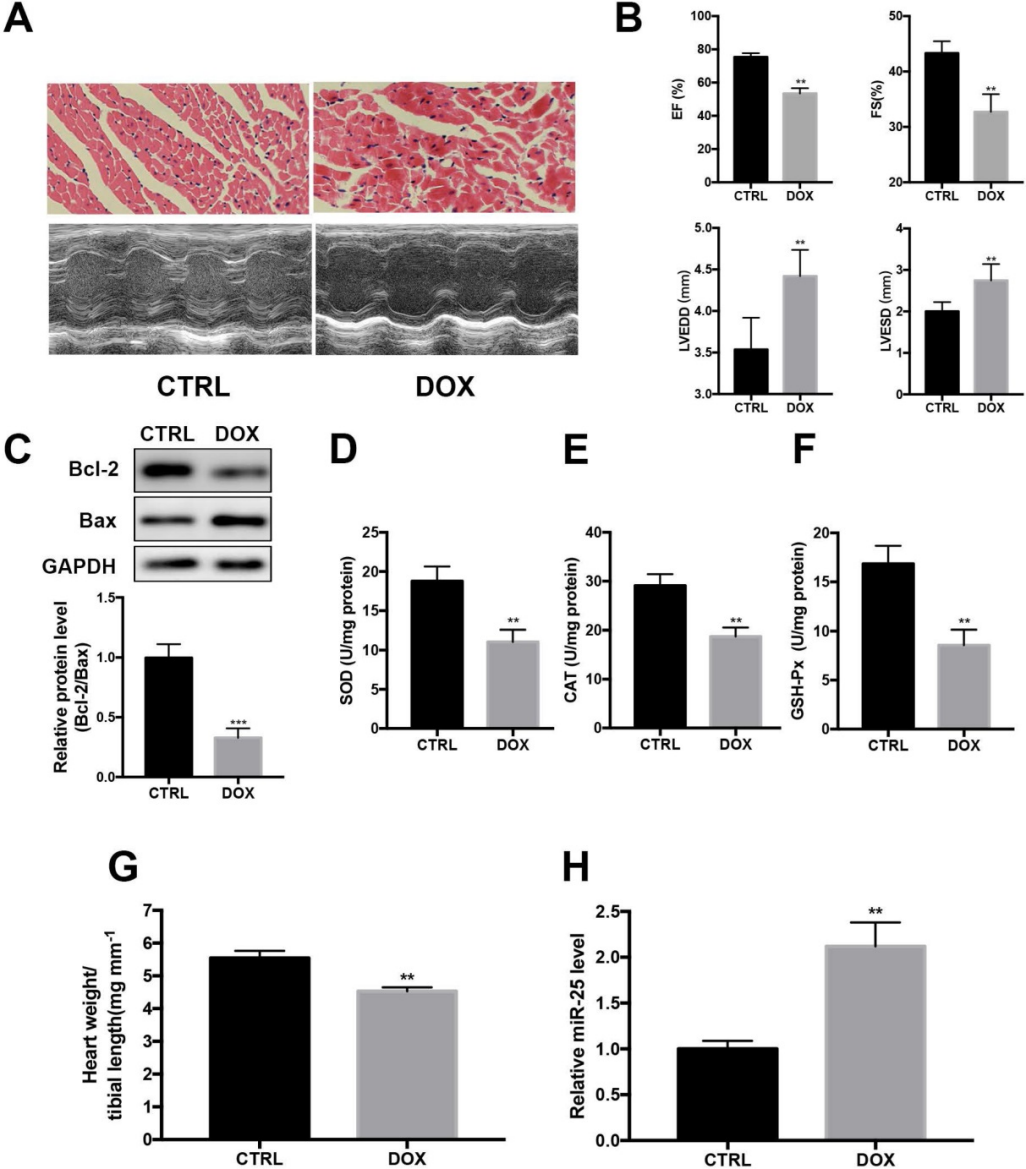
** miR-25 expression is augmented after treatment of DOX in mice.** (A) After stimulated with DOX or saline for 4 weeks, representative images of H&E staining from mice (upper panel, scale bar=50μm) and representative M-mode echocardiography of left ventricular chamber change (lower panel). Left ventricular performance was measured in mice and the variables (left ventricular ejection fraction (EF), fraction shortness (FS) left ventricular end-diastolic dimension (LVEDD) and end-systolic dimension (LVESD) between different treatment groups are shown in (B). (C) DOX decreases the protein level of Bcl-2, whereas increases the level of Bax in heart tissue. Antioxidant enzyme activities of SOD, CAT and GSH-Px are reduced by DOX (D-F). (G) DOX decreases the heart weight to tibial length ratio compared with control group in mice. (H)The expression of miR-25 in mice after treated with DOX. (*P < 0.05; **P < 0.01; ***P < 0.001, n=7)

Next, we investigated whether DOX regulated the expression of miR-25 in DOX-treated mice. HE staining indicated that DOX treatment resulted in the disturbance of cardiac tissue structure (Figure 2A). Echocardiography showed that DOX induced markedly left ventricular contractile function indicated by the decreased EF and FS and increased of LVEDD and LVESD (Figure 2A and B). Moreover, the protein level of Bcl-2 was decreased with the level Bax increased in DOX group (Figure 2C). The activities of SOD, CAT and GSH-Px, which are important intracellular antioxidant enzymes, were also tested in heart tissue. As a result, compared with the CTRL group, activities of SOD, CAT and GSH-Px were decreased in DOX group, indicating increased oxidative stress in the heart after treated with DOX (Figure 2D-F). In addition, the heart-to-tibial-length ratio of the DOX group was decreased, indicating the myocardial atrophy induced by DOX (Figure 2G). Consistent with *in vitro* result, cardiac level of miR-25 also showed an increase compared with the control group (Figure 2H). Taken together, these results indicated that miR-25 expression was involved in DOX-induced cardiac injury.

### miR-25 regulates DOX-induced apoptosis

**Figure 3:**
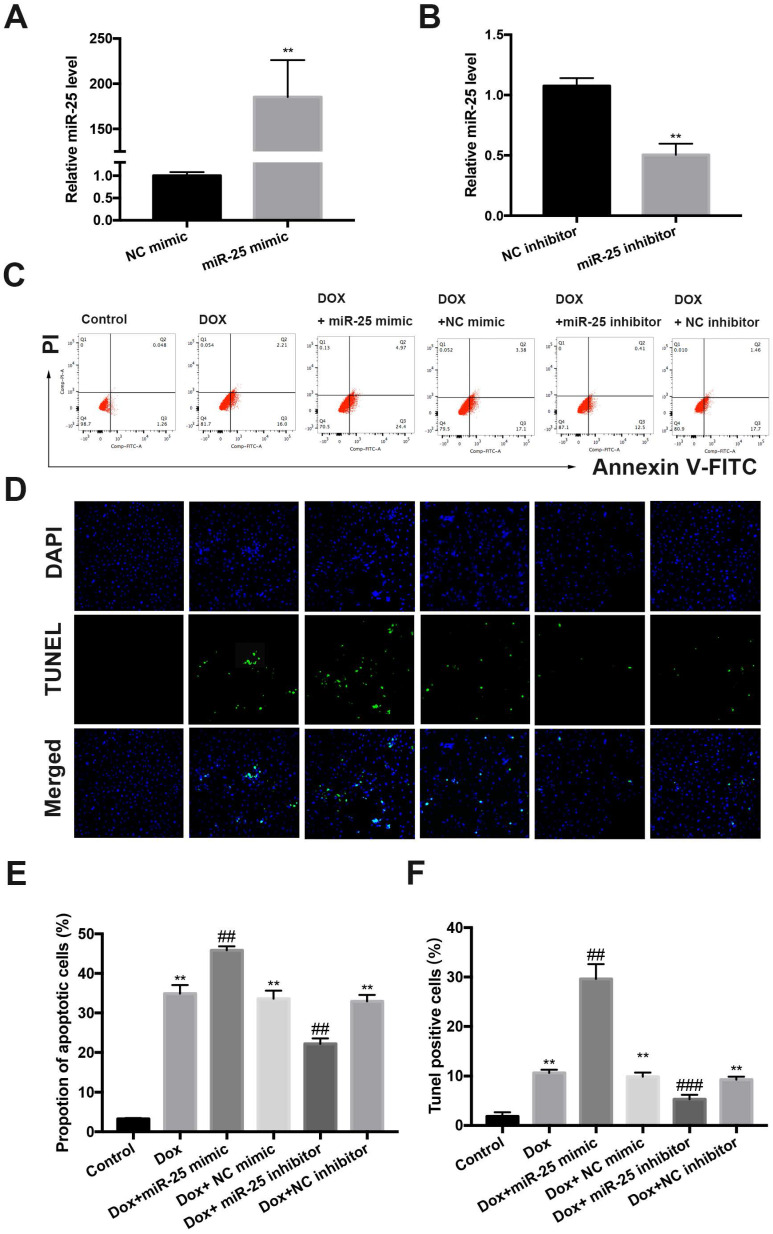
** The protective effect of miR-25 inhibition in DOX-induced apoptosis.** The relative expression of miR-25 after transfection of miR-25 mimic(A) and miR-25 inhibitor(B) (**P < 0.01, n=4). (C) Representative dot plots of flow cytometric images in H9c2 cells after treated as indicated. (D)TUNEL assay results of different groups (original magnification ×400). Nuclei are stained in blue, and TUNEL staining is shown in green. Quantification of the results in C (E), and D (F). (*P < 0.05; **P < 0.01; ***P < 0.001 compared with control group; ^#^p < 0.05; ^##^p < 0.01 compared with DOX+NC-treated group, n = 5)

To investigate the role of miR-25 in DOX-induced cell apoptosis, which is a hallmark in DOX-induced cardiac injury, we established a cell culture model of inhibition or overexpression miR-25 using miR-25 inhibitor or miR-25 mimic. Transfection of cells with miR-25 mimic or inhibitor induced the miR-25 levels highly increased in the miR-25 mimic group or significantly decreased in the miR-25 inhibitor group compared with the control group by RT-qPCR (Figure 3A and 3B). DOX treatment for 24 hours resulted elevated apoptosis in H9c2 cells. Both flow cytometry analysis and (Figure 3C and 3E) TdT-mediated dUTP nick end labeling (TUNEL) staining (Figure 3D and 3F) indicated that the miR-25 mimic significantly exaggerated DOX-induced apoptosis, whereas inhibition of miR-25 decrease the rate of apoptotic cells. Western blot revealed that DOX induced a higher level of Bax, but a lower level of Bcl-2, and miR-25 mimic led to further increase of Bax and decreased Bcl-2 expression. By contrast, miR-25 inhibitor confirmed the opposite effects on the abovementioned proteins (Supplement Figure 1 A and B). These data suggest that increased miR-25 expression might contribute to DOX-induced apoptosis.

### Inhibition of miR-25 leads to decreased ROS and DNA damage

**Figure 4:**
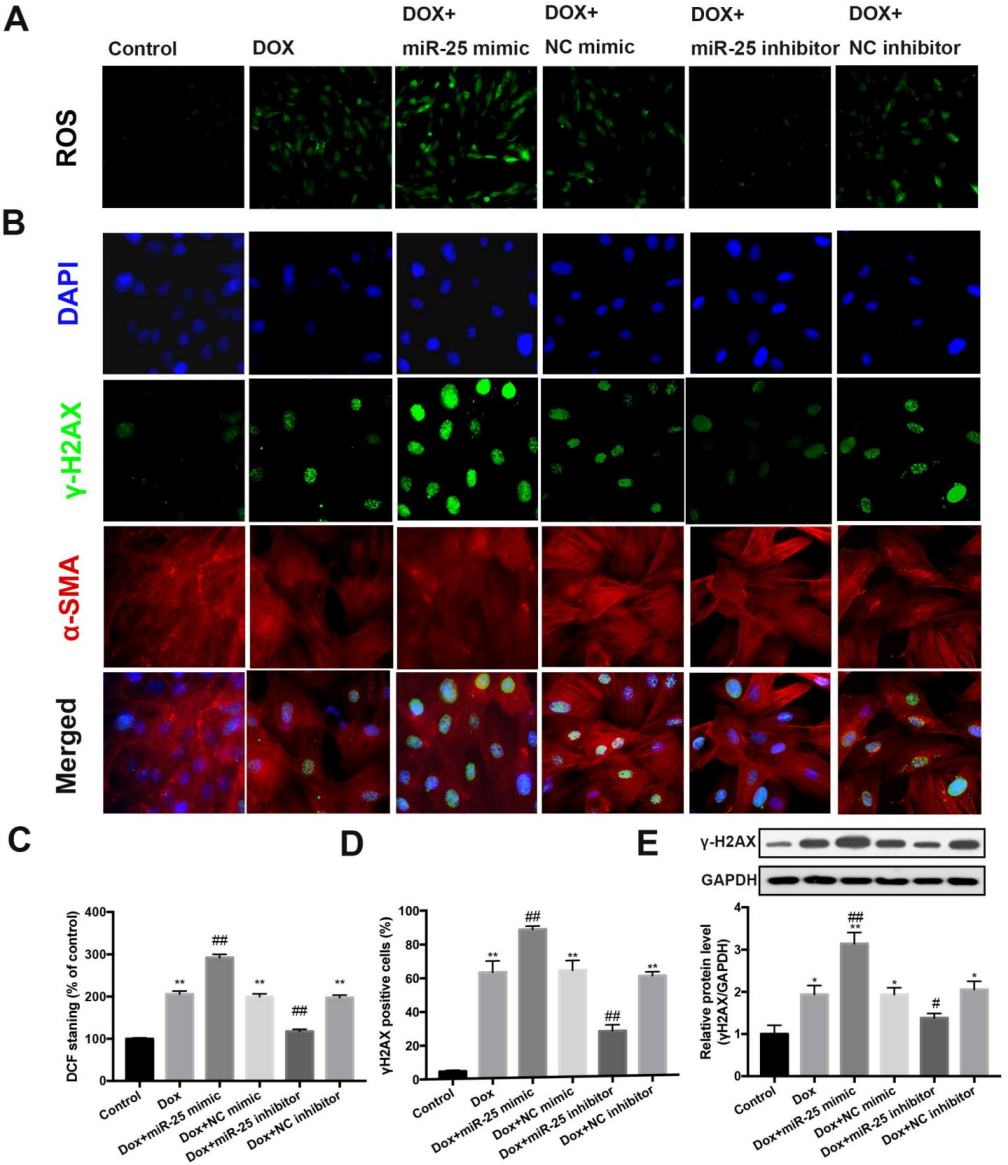
** miR-25 mediates ROS production and DNA damage process.** (A) Intracellular reactive oxygen species (ROS) level in DOX-treated H9c2 cells with transfection of miR-25 inhibitor or miR-25 mimic. (B) The image shows the expression level of γ-H2AX in DOX-treated H9c2 cells with transfection of miR-25 inhibitor or miR-25 mimic. Nuclei are stained in blue, cytoskeleton in red and γ-H2AX in green. The bar graphs show the percentages of ROS levels (C) and γ-H2AX-positive cardiomyocytes of each group (D). (*P < 0.05; **P < 0.01; ***P < 0.001 compared with control group; ^#^p < 0.05; ^##^p < 0.01 compared with DOX group, n = 5). (E) Parallel gels were run for γ-H2AX protein and GAPDH, under the gels are the quantification results. (*P < 0.05; **P < 0.01; ***P < 0.001 compared with control group; ^#^p < 0.05; ^##^p < 0.01 compared with DOX group, n = 3)

Previous studies reported oxidative stress could induce the overexpression of miR-25 22, 23. To this end, we evaluated whether miR-25 could reduce the production of ROS. Notably, assessment of ROS fluorescence in cells determined that inhibition of miR-25 results in a marked reduction in ROS fluorescence induced by DOX, whereas miR-25 mimic exhibited the contrary result (Figure 4A and 4C). Next, we evaluated if miR-25 inhibitor alleviated DOX-induced DNA damage, which might due to the direct or indirect ROS production. We performed immunostaining to examine the formation of γ-H2AX nuclear foci (a DNA damage marker 24). Consistent with the excessive oxidative stress, treated H9c2 cells with DOX for 24h increased the γH2AX nuclear foci and γ-H2AX-positive cells, and this effect was counteracted by miR-25 inhibitor, compared with that in the control cells. In contrary, the γ-H2AX nuclear foci generation and number of γ-H2AX-positive cells was significantly increased in enforced miR-25 expression cells. (Figure 4B and 4D). In addition, western blot analysis of γ-H2AX expression also showed similar results (Figure 4E). Based on these data, we can confirm that miR-25 facilitated DOX-induced ROS and γ-H2AX formation.

### PTEN is a target of miR-25

**Figure 5:**
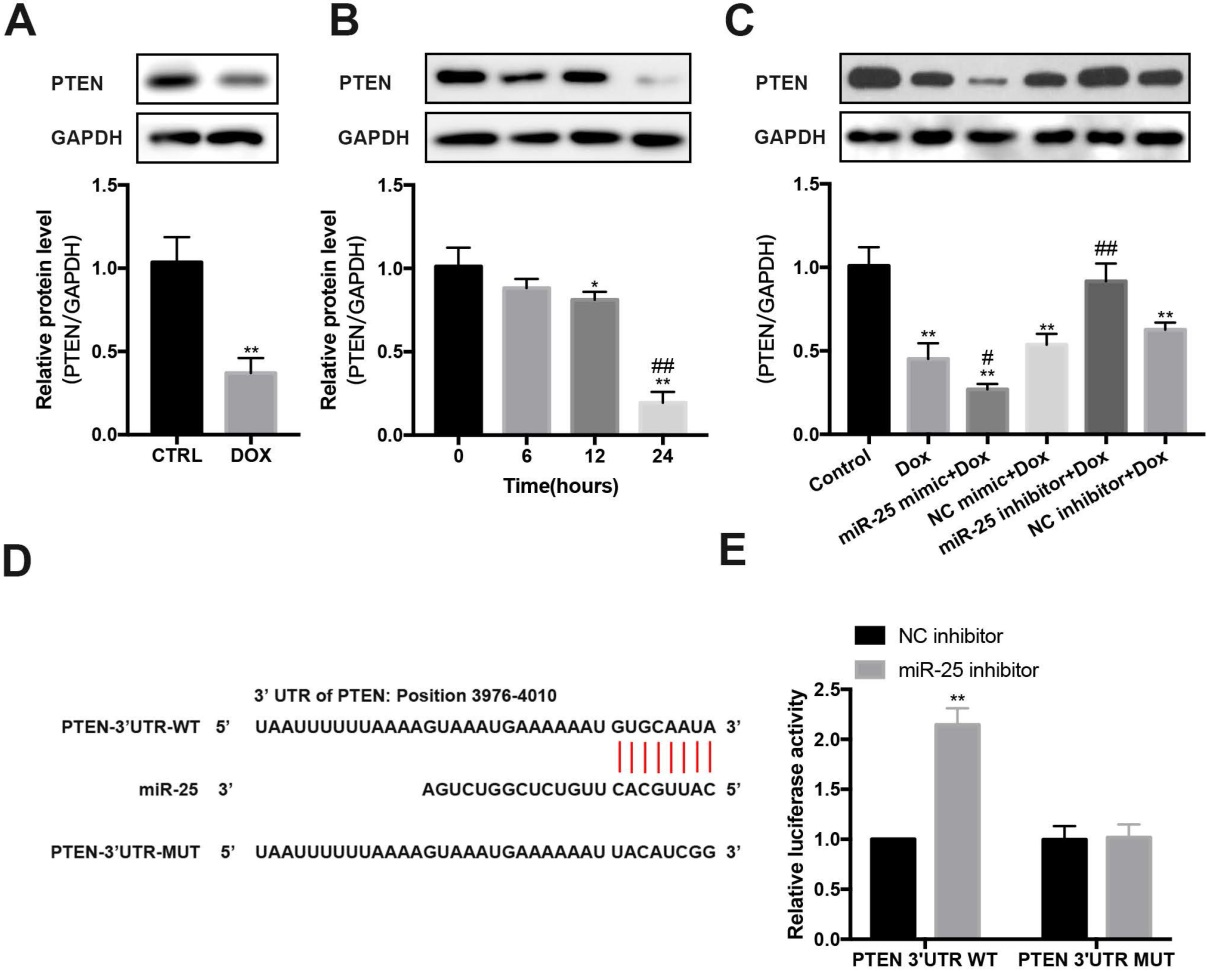
** miR-25 regulates the expression of PTEN.** (A) Relative expression of PTEN measured from mice heart tissue lysates treated with DOX or CTRL. (B) After exposure to 5μM DOX for 6, 12, 24, 48h, PTEN levels were determined by Western blot analysis. (*P < 0.05; **P < 0.01 compared with cells in 0h; ^##^p < 0.01 compared with cells in 6h, n = 3) (C) After transfection of miR-25/NC mimic or miR-25/NC inhibitor, H9c2 cells were treated with 5μM DOX or control, and the PTEN expression was measured. GAPDH was served as the loading control. (*P < 0.05; **P < 0.01 compared with control group; ^#^p < 0.05; ^##^p < 0.01 compared with DOX group, n = 3) (D) Sequence alignment of wild-type (WT) PTEN and mutated (MUT) PTEN mRNA 3′-UTR binding site of miR-25. (E) Luciferase activity levels upon co-transfection of a luciferase construct containing PTEN-3′UTR-WT or PTEN-3′UTR-MUT with miR-25 mimic or NC mimic in HEK293T cells. (**P < 0.01, n = 3)

In DOX-induced cardiomyopathy mice model, we found that PTEN protein level was significantly decreased (Figure 5A). Furthermore, after exposing to DOX for 0, 6, 12 or 24 hours, the expression of PTEN reduced in a time-dependent manner in H9c2 cells (Figure 5B). Then we test whether miR-25 can control PTEN expression. Results showed that enforced expression of miR-25 could decrease the level of PTEN, whereas knockdown of miR-25 resulted in an elevation expression of PTEN (Figure 5C). To explore whether miR-25 regulates the expression of PTEN through a direct or indirect manner, we analyzed the 3′-untranslated region (UTR) sequence of PTEN via bioinformatics. The binding sites between miR-25 and PTEN 3′UTR was shown in Figure 5D. Previous studies also determined that PTEN is a target of miR-25-3p 25-27. To validate the target reaction between miR-25 and PTEN, we co-transfected the PTEN-WT and miR-25 inhibitor/NC inhibitor compared with co-transfection of PTEN-MUT and miR-25 inhibitor/NC inhibitor. After twenty-four hour's transfection, in wild reporter plasmid was remarkably repressed by miR-25 inhibitor compared with NC inhibitor, whereas this effect was not observed with the PTEN-MUT group (Figure 5E). These results indicate that miR-25 regulates the expression of PTEN.

### Inhibition of PTEN blocks the beneficial of miR-25 suppression

**Figure 6:**
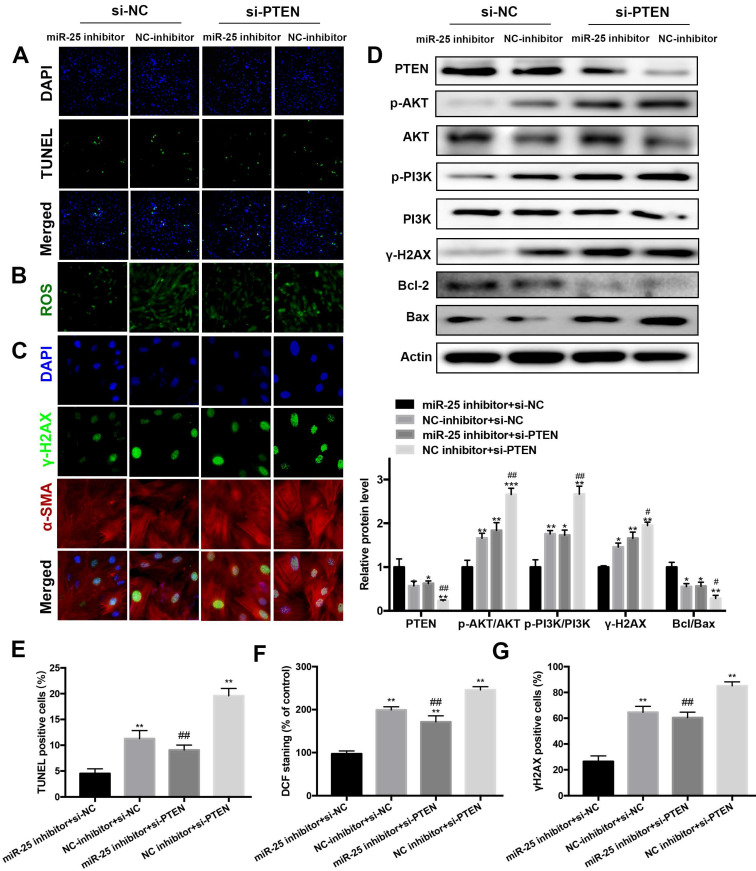
** Silencing of PTEN blunts the protective effect of miR-25 inhibition.** H9c2 cells were co-transfected with miR-25 inhibitor or NC inhibitor and si-PTEN or si-NC (5μM) and then stimulated with DOX (5μm) for 24 h. Then, the TUNEL stainin (A), ROS (B) and γ-H2AX (C) levels in control and PTEN knockdown cells were measured. (D) Protein expression levels of PTEN, p-AKT, AKT, p-PI3K, PI3K, γ-H2AX, Bcl-2, and Bax in H9c2 cells were determined by Western blot analysis and quantification of the relative expression using Actin as an internal control (lower panel). (*p < 0.05, ** p < 0.01, *** p < 0.001, compared with the miR-25 inhibitor + si-NC group, ^#^p < 0.05,^ ##^p < 0.01,^ ###^p < 0.001, compared with the NC inhibitor + si-NC group. n = 3). Quantification of the results in A (E), B (F), and C(G). (*p < 0.05, ** p < 0.01, *** p < 0.001, compared with the miR-25 inhibitor + si-NC group, ^#^p < 0.05,^ ##^p < 0.01, ^###^p < 0.001, compared with the NC inhibitor + si-NC group. n = 5)

To elucidate whether PTEN was responsible for the antiapoptotic and DNA damage effects of miR-25 inhibition, PTEN was downregulated by transfection with PTEN interfering RNA (siRNA). In DOX-treated cells, the protein level of PTEN was significantly increased in the miR-25 inhibitor+ PTEN siRNA group as compared with the miR-25 inhibitor + NC siRNA group (Figure 6D). Moreover, the results of TUNEL staining and Western blot analysis of Bcl-2/Bax ratio showed that PTEN siRNA blocked the anti-apoptotic effect of miR-25 inhibitor (Figure 6A, 6D and 6E). In line with the above results, knockdown of PTEN also blunter the effects of miR-25 inhibitor in reduction of ROS generation and DNA damage (Figure 6B, 6C, 6F and 6G). It is confirmed the activation of PI3K and AKT are modulated by PTEN, so we investigated the effect of miR-25 on the PI3K/AKT signaling pathway. After treating with DOX and miR-25 inhibition, the phosphorylation level of PI3K and AKT were both reduced compared with NC inhibition group, whereas this effect was reversed after transfection with PTEN siRNA (Figure 6D). Taken together, inhibition of PTEN activation reversed miR-25 inhibitor-induced protection against DOX-induced cardiotoxicity via PI3K/AKT pathway.

### Inhibition of miR-25 does not influence the anti-cancer ability of DOX

**Figure 7:**
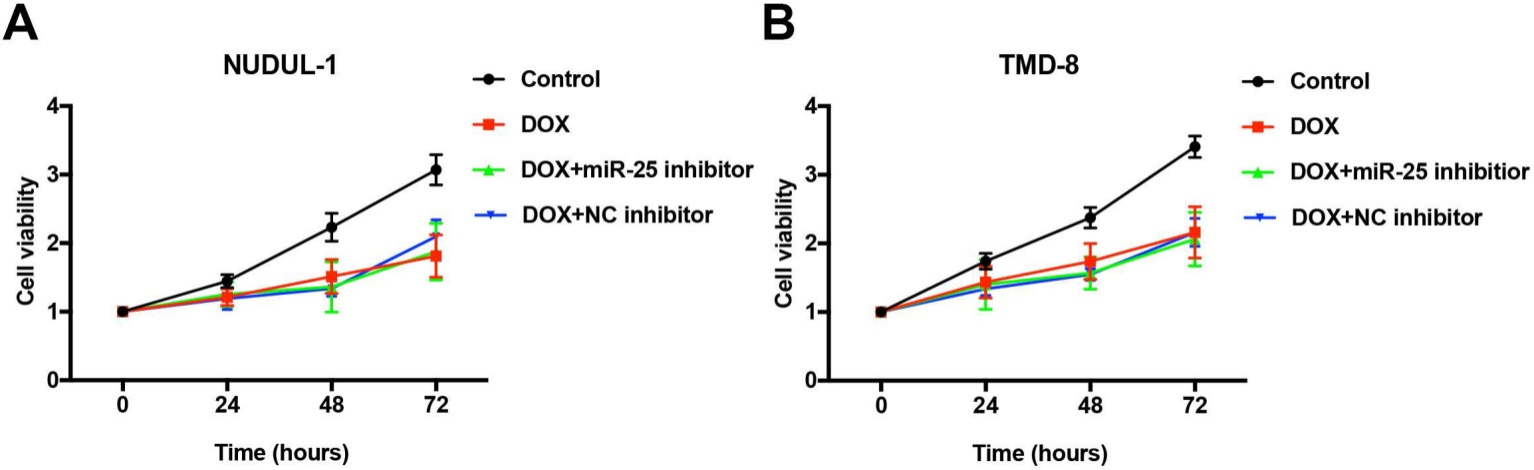
** miR-25 inhibition exerts no effect on DOX treatment in DLBCL cells.** After NUDUL-1 and TMD8 cells were co-transfected with miR-25 inhibitor or NC inhibitor, DOX (5μm) was added into the cells for 24, 48, 72 and 96 h. CCK-8 results show the cell viability changes in NUDUL-1(A) and TMD8(B) cells.

Concerning that DOX is an essential first-line drug used in treating DLBCL patients (rituximab, cyclophosphamide, doxorubicin, vincristine, and prednisone, R-CHOP) 4, we tested whether the effects of miR-25 changed the anti-tumor ability of DOX in DLBCL cells. As the results of CCK-8 show, after treated with 5μM DOX for 24, 48, 72 or 96 hours, the cell abilities of NUDUL-1 and TMD8 were significantly decreased. However, the inhibition of miR-25 expression does not comprise the cytotoxicity of DOX in DLBCL cells (Figure 7 A and 7B). These results indicate that miR-25 inhibition might have little effect on the treatment of DOX in cancers.

## Discussion

DOX, one of the frequently used anthracyclines in treating tumors, is known to exert dose-dependent cardiotoxicity in the long-term. Even rodent efforts were made to reduce DOX-induced cardiotoxicity, there still exist many patients remain incurable and die for heart failure 28, 29. The major mechanism of DOX-induced cardiotoxicity is free radical generation resulting in DNA damage and cell apoptosis 30, 31. In the present study, we revealed a new mechanism for the protective effect of inhibition miR-25 on DOX-induced cardiotoxicity. DOX treatment upregulated the expression of miR-25 in DOX-treated mice and H9c2 cells, and inhibition of miR-25 protected H9c2 cells against DOX-induced apoptosis, ROS generation and DNA damage via upregulating the expression of PTEN and thus resulting the decreased expression of p-PI3K and p-AKT.

Previous studies have described the role of miRNA in the pathological processes of DOX-induced cardiomyopathy. For instance, Shashi Kumar Gupta et al.^32^ showed that overexpression of the miR-212/ 132 family using adeno-associated virus (AAV) could alleviate the development of DOX-induced cardiotoxicity by targeting fat storage-inducing transmembrane protein 2 (Fitm2). Downregulation of Nrf2 and Sirt2 by miR-140-5p showed an increased DOX-induced myocardial oxidative damage 13. However, the significance of many miRNAs in DOX-induced cardiotoxicity is still not well understood. Given miR-25 plays an apical role in the process of heart failure and elevated expression of miR-106b~25 cluster is related to doxorubicin-induced senescence 33, we hypothesized that miR-25 might involve in the process of DOX-induced heart failure. We determined that the expression of miR-25 was increased after exposure to DOX both *in vivo* and* in vitro*. Accumulating studies has determined that the most prominent features of DOX-induced cardiotoxicity are cell apoptosis and excessive oxidative stress. In our current study, inhibition of miR-25 remarkably suppressed the cell apoptosis and ROS generation. Conversely, overexpression of miR-25 using miR-25 mimic aggravated these results. We also accessed the influence of miR-25 on apoptotic related protein, Bax and Bcl-2, to figure out the mechanism of anti-apoptosis effect of miR-25. We found that the silencing of miR-25 increased the protein level of Bcl-2 and downregulated the level of Bax. DNA damage is another hallmark of DOX-induced cardiotoxicity, which could also partly due to the production of ROS. H2AX, known as a variant of H2A, plays an apical role in DNA repair/damage resulting in the formation of phosphorylated histone H2AX (γ-H2AX) 34, 35. The results of western blot and immunofluorescence of this marker both determined that miR-25 inhibitor could alleviate DNA damage.

**Figure 8:**
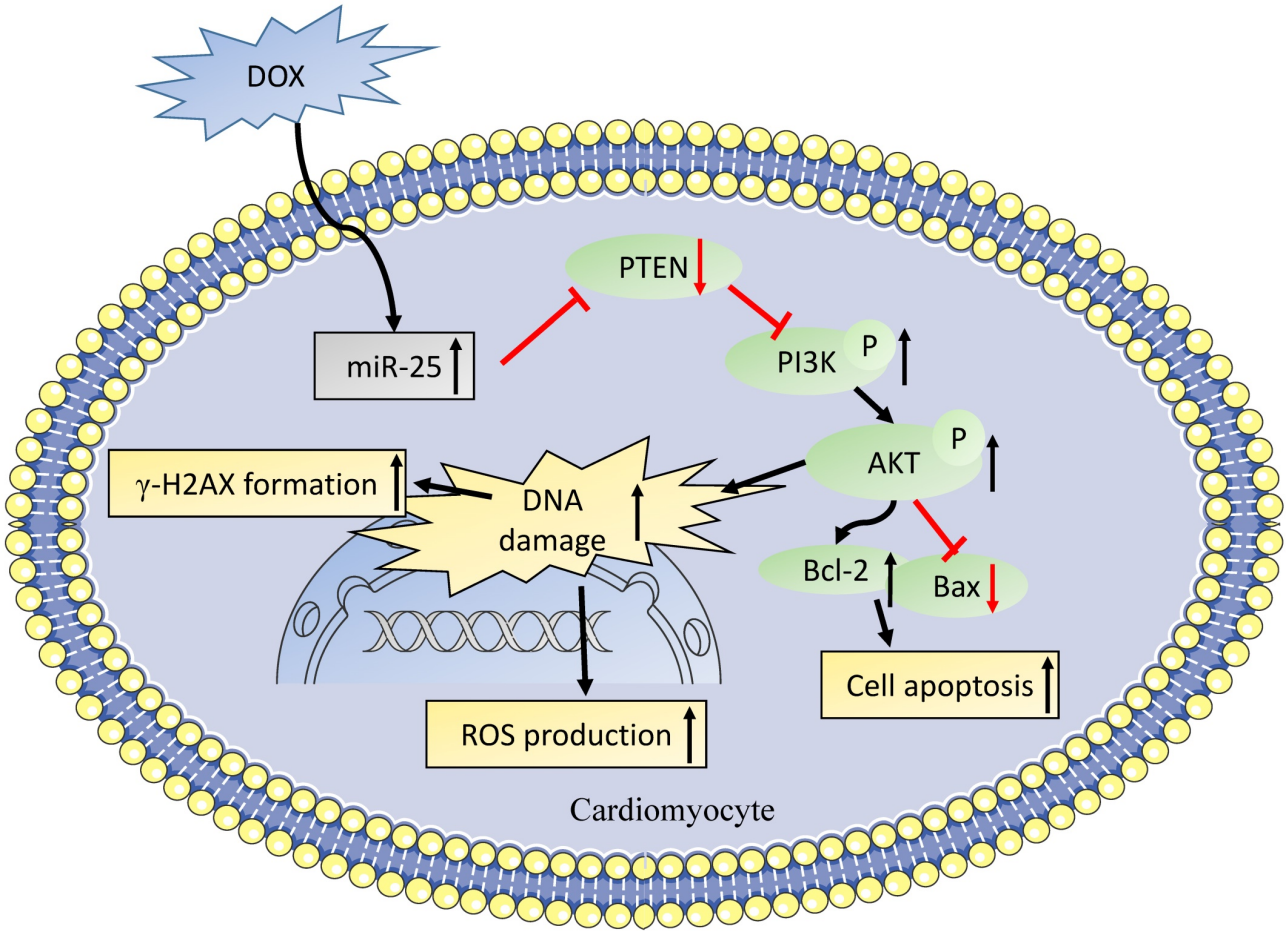
A role of miR-25 in DOX-induced cardiotoxicity through PTEN/PI3K/AKT pathway.

PTEN, a dual lipid/protein phosphatase, is identified as a tumor suppressor, which also regulates many critical process in the development of cardiovascular diseases 36. In the cardiac muscle specific knockout of PTEN mice model, the loss of PTEN resulted in reduced cardiac contractility and increased cell size 37, which might be attributed to the suppression of Pink1 and AMPK phosphorylation in the heart 38. It was also reported that PTEN was involved in DNA repair, which was regulated by the expression of ATM to reduce the γ-H2AX foci 39, which is line with our results. Moreover, it was also showed that PTEN deletion in Ishikawa cells increased DOX-caused apoptosis 40. Consistent with previous studies, we observed the level of miR-25 was elevated in a time- dependent manner, while as western blot result showed that PTEN was gradually decreased after exposing to DOX for 0, 6, 12, or 24h. We also confirmed that PTEN was a direct target of miR-25 by luciferase reporter assay. After transfection with siRNA of PTEN, the expression of PTEN was significantly decreased. When co-transfection with miR-25 inhibitor, PTEN suppression partially blunted the function of miR-25 inhibitor in protection against the DOX-induced cardiotoxicity. A recent study from Yihua Bei et al. 41 also shown miR-21 knockout mice exhibited anti-aging effect via increasing PTEN level. Moreover, it was identified that the HER2 inhibitor lapatinib increased the cardiotoxicity induced by DOX via promoting PI3K and AKT phosphorylation, whereas the iNOS inhibition reversed this activation 42. Our study also indicated that the inhibition of miR-25 downregulated the expression of p-PI3K and p-AKT, which in turn led to an increase in Bcl-2/Bax ratio (Figure 8).

Concerning that the first purpose of DOX treatment is to increase cancer patient's survival, it is important to find a suitable therapy that could not only alleviate DOX-induced heart injury, but also with no effect on the progression of tumors. Previous studies described that elevated of miR-25 induce the progression of cancers 43, 44, whereas our results showed inhibition of miR-25 decreased DOX related cell apoptosis, ROS production and DNA damage, it might be a promising therapy method towards DOX-induced cardiotoxicity. Given DOX is frequently used in treating DLBCL, our results also confirmed that miR-25 inhibition did not change the anti-cancer ability of DOX in DLBCL cells. The possible mechanism might be due to that PTEN mutant is very common in DLBCL patients^45^, which results in the effect of miR-25 by binding with PTEN disappeared. Future studies should focus on developing a more stable manner to deliver it into human body, such as Tough Decoy inhibitor and so on 46.

In summary, we identified that miR-25 inhibition exerted a protective role in DOX-induced injury in H9c2 cell, which might partially by mediating PTEN/PI3K/AKT signaling pathway. These findings allow us to better understand the role of miR-25 in the pathological process of DOX-induced cardiomyopathy and ultimately develop potential protective strategy for these patients.

## Supplementary Material

Supplementary figures and tables.
