# Supplementary material for: Inhibition of miR-25 attenuates doxorubicin-induced apoptosis, reactive oxygen species production and DNA damage by targeting PTEN
Source: Int J Med Sci. 2020 Jun 5;17(10):1415–27. doi: 10.7150/ijms.41980 (PMC7330660; doi:10.7150/ijms.41980)

Supplement Figure 1 miR-25 modulates apoptotic associated protein expression

(A)Immunoblotting showing that miR-25 overexpression prevented the DOX-induced increasing expression of Bax and decreasing expression of Bcl-2, whereas miR-25 repression exhibited contrast results. (B) Quantification of the relative results of Bax and Bcl-2 expression. (*p < 0.05, ** p < 0.01, compared with the control group, #p < 0.05, compared with DOX group. n = 3)

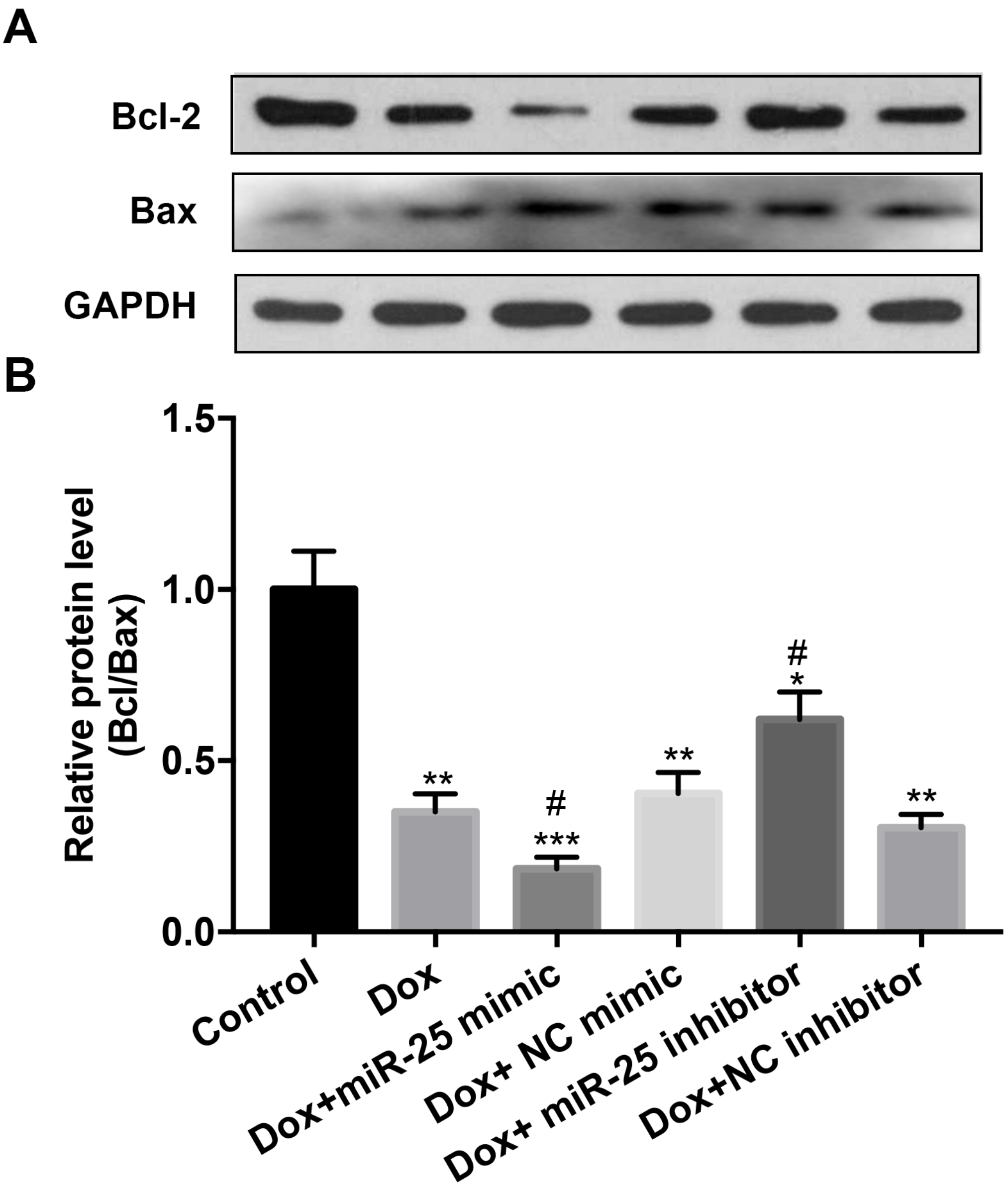

Supplement: Supplementary file 1 — Supplementary figures and tables. [file ijmsv17p1415s1.pdf]
